# Prognostic Value of Relevant Clinicopathologic Variables in Epithelioid Sarcoma: A Multi-Institutional Retrospective Study of 44 Patients

**DOI:** 10.1245/s10434-014-4294-1

**Published:** 2015-02-07

**Authors:** Naofumi Asano, Akihiko Yoshida, Koichi Ogura, Eisuke Kobayashi, Michiro Susa, Hideo Morioka, Shintaro Iwata, Takeshi Ishii, Toru Hiruma, Hirokazu Chuman, Akira Kawai

**Affiliations:** 1 Department of Musculoskeletal Oncology, National Cancer Center Hospital, Tokyo, Japan; 2 Department of Pathology, National Cancer Center Hospital, Tokyo, Japan; 3 Department of Orthopaedic Surgery, Keio University School of Medicine, Tokyo, Japan; 4 Division of Orthopaedic Surgery, Chiba Cancer Center, Chiba, Japan; 5 Division of Musculoskeletal Tumor Surgery, Kanagawa Cancer Center, Yokohama, Japan

## Abstract

**Background:**

Epithelioid sarcoma (ES) is an extremely rare soft tissue sarcoma. Recently, the proximal variant has been reported to be a more aggressive subtype; however, as most reports of ES have involved small case series, the actual prognostic implications remain unclear. We investigated the clinicopathological features of patients with ES to identify the prognostic factors that influence survival.

**Methods:**

We retrospectively analyzed the clinicopathological features of 44 patients with ES who had been treated at our institutions between 1991 and 2011. Among these patients, 26 were diagnosed histologically as having classic-type ES, whereas the remaining 18 had proximal-type ES. Thirty-three of the patients, all without distant metastases, underwent curative surgery, and the remaining 11 with distant metastases (M1) received palliative treatment.

**Results:**

The proximal subtype was significantly correlated with a proximal tumor location, distant metastases at presentation, presence of rhabdoid cells, a higher tumor grade, and vascular invasion. The overall survival (OS) rate at 5 years for the 44 patients was 45 %. A superficial tumor location and lymph node metastases (N1) at presentation were independently predictive of local recurrence-free survival (LRFS), and N1 and M1 tumors were independently predictive of distant metastasis-free survival and OS, respectively. The proximal subtype was associated with unfavorable LRFS and OS, although not to a statistically significant degree.

**Conclusions:**

Proximal-type ES has significantly more aggressive clinicopathological features than classic-type ES, and lymph node or distant metastasis has the most critical impact on prognosis.

Epithelioid sarcoma (ES) is an uncommon malignant soft tissue tumor comprising approximately 1 % of all soft tissue sarcomas. It is a slow-growing tumor occurring mainly in young adult males, predominantly affecting subcutaneous tissues, fasciae, or tendon sheaths of the extremities, with a predilection for the hands and forearms.^1^–^3^

Histologically, the classic type shows a distinctive nodular, granuloma-like pattern, with spindle and epithelioid cells circumscribing areas of central degeneration and necrosis.^4^,^5^ However, a subtype of ES, the proximal type, occurs mostly in deep-seated soft tissues in the truncal region and is characterized histologically by sheets of large, atypical, epithelioid cells with vesicular nuclei and prominent nucleoli, showing a rhabdoid phenotype.^6^,^7^

Unfavorable prognostic factors for overall survival (OS) have been reported to include a proximal location.^2^,^8^,^9^ Recently, several authors have reported that the proximal subtype has a poor prognosis.^6^,^7^,^9^–^11^ However, as most previous reports have involved only small case series, especially with regard to proximal-type ES, the true prognostic impact of the ES subtype remains unclear.^9^,^12^

In the present study, we therefore investigated the prognostic value of relevant clinicopathologic variables in 44 patients with ES.

## Materials and Methods

### Patients

We reviewed a prospective database for four institutions (Higashi-nihon Orthopaedic and Pediatric Sarcoma Group; HOPES) covering the period from 1991 to 2011. A total of 44 patients with a confirmed diagnosis of ES made by a specialized pathologist (AY) were analyzed. The median follow-up period was 26.5 (range 1–168) months overall. All patients or their guardians provided informed consent, according to the rules approved by the respective Institutional Review Boards.

### Diagnostic Criteria for Each Histological Subtype

The diagnostic criteria for ES were the same as those documented previously.^6^ Histologically, classic-type ES commonly presents as a multinodular proliferation of eosinophilic, epithelioid, and spindle-shaped cells. Usually, the lesion shows minimal cytologic atypia, with vesicular nuclei and often single and central nucleoli. Occasionally, the epithelioid cells may have a rhabdoid appearance, and tumor nodules may frequently undergo central necrosis, resulting in a pattern resembling a benign necrobiotic granulomatous process. Proximal-type ES is characterized by a predominantly large epithelioid cytomorphology, marked cytologic atypia, frequent occurrence of rhabdoid features, and lack of a granuloma-like pattern in most cases. ES with hybrid features of both classic and proximal-type ES is grouped as proximal-type ES.

### Study Parameters

At the time of diagnosis, local tumor extent was assessed using computed tomography (CT) and/or magnetic resonance imaging (MRI). Lymph node and/or distant metastasis before treatment was assessed using enhanced CT and/or whole-body positron emission tomography (PET)-CT. Tumor location in the distal extremity was defined as ‘distal’, and localization in the proximal extremity or axial trunk was defined as ‘proximal’. Disease stage was classified using the American Joint Committee on Cancer/International Union Against Cancer TNM staging system version 7 (AJCC stage).^13^ Histological grade was assessed using the French Federation of Cancer Centers (FNCLCC) grading system.^14^

### Treatment

Thirty-three patients without distant metastasis at presentation (M0) underwent surgical treatment of the primary tumor with curative intent, which comprised wide resection in 14 patients, additional wide resection in 9 patients, and amputation in 10 patients. Regional lymphadenectomy was performed in 12 patients who had regional lymph node swelling. Six of these 12 patients were found to have regional lymph node metastases, and the remaining 6 were found to be negative after histological examination. The surgical margins were microscopically negative in 24 (73 %) patients, positive in 8 (24 %) patients, and not available (NA) in one patient. Adjuvant therapy included preoperative radiotherapy in 2 patients (6 %), postoperative radiotherapy in 4 (12 %) patients, pre-/postoperative systemic anthracycline-based chemotherapy in 1 patients (3 %), and postoperative chemotherapy in 2 patients (6 %).

Eleven patients (25 %) with distant metastasis at presentation (M1) received palliative treatment with various types of chemotherapy consisting of doxorubicin and/or ifosfamide, radiotherapy, and/or surgery.

### Statistical Analysis

In order to compare the differences in clinicopathological features between classic- and proximal-type ES, the *χ* ^2^ test was employed. Local recurrence-free survival (LRFS), distant metastasis-free survival (DMFS), and OS were calculated from the clinical databases using the Kaplan–Meier method. Patients with M0 tumors were evaluated for LRFS, DMFS, and OS from the date of definitive surgery until the most recent follow-up, recurrence, or death. All patients were evaluated for OS from the date of diagnosis at our institutions until the most recent follow-up or death. The log-rank test was used to compare the survival curves for the different subgroups of patients to establish the potential prognostic value of various factors. Stepwise multivariate Cox regression analyses were performed to identify prognostic factors that were significant. Prognostic factors with statistical significance (*p* < 0.05) in the univariate analysis were included in the multivariate analysis. Statistical analysis was performed using the PASW statistics 18 package (SPSS Inc., Chicago, IL, USA).

## Results

### Clinical Features

Thirty patients (68 %) were male and 14 (32 %) were female. The median age at the first visit to our hospitals was 37 (range 6–69) years. Twenty tumors (46 %) were located in distal extremities (forearm, seven; hand, four; finger, five; lower leg, one; foot, three), eight (18 %) in proximal extremities (upper arm, one; axilla, two; buttock, two; thigh, three) and 16 (36 %) in the trunk (paravertebral region, two; back, two; abdominal wall, two; inguinal region, five; perineum, five;). Thirty-one tumors (70 %) were deep-seated, 12 (27 %) were superficial, and 1 (3 %) was not evaluable. Seven of 12 superficial tumors occurred in the inguinal or perineal region. The median tumor size was 5 (range 1–17) cm; 25 lesions (57 %) were primary, whereas 19 (43 %) were recurrent. On the basis of AJCC staging, 18 cases were stage II, 12 were stage III (including 6 with N1M0), 11 were stage IV (5 with N1M1), and 3 were not evaluable.

### Histological Features

FNCLCC grade was assessed in all 44 cases: 32 cases (73 %) were grade 2, and the remaining 12 (27 %) were grade 3. Twenty-eight cases (64 %) had hemorrhage, 12 (27 %) had vascular invasion, 6 (14 %) had perineural invasion, and 6 (14 %) had reactive bone formation.

Immunohistochemical studies showed that pan cytokeratin AE1/AE3 reactivity was present in all 44 cases. CD34 was positive in 28 (74 %) of 38 cases that were examined, and S-100 was minimally positive in 4 (14 %) of 24 cases that were examined. Loss of expression of INI1 was evident in all (100 %) of the 29 cases where this was examined.

Twenty-six cases (60 %) were diagnosed as classic-type ES and 18 (40 %) were diagnosed as proximal-type ES. Comparisons between the two histologic types revealed that the proximal type was significantly correlated with a proximal location (*p* < 0.001), distant metastasis at presentation [M1] (*p* = 0.031), presence of rhabdoid cells (*p* = 0.002), a higher tumor grade (*p* = 0.045), and vascular invasion (*p* = 0.007) (Table 1). Proximal-type ES also showed a higher incidence of N1 disease (proximal-type 39 % vs. classic-type 15 %), although statistically this was not significant (*p* = 0.093) (Table 1).

**Table 1:** Various clinicopathological variables in cases of classic- and proximal-type epithelioid sarcoma

Variable	Total (*n*)	Classic type (*n*)	Proximal type (*n*)	*p* Value
Sex				1.000
Male	30	18	12	
Female	14	8	6	
Age at presentation (years)				0.761
≤30	16	10	6	
>30	28	16	12	
Location				<0.001
Distal	20	19	1	
Proximal	24	7	17	
Depth (*N* = 43)				0.168
Superficial	12	5	7	
Deep	31	21	10	
Tumor size (cm) (*N* = 39)				0.209
≤5	22	15	7	
>5	17	8	9	
Stage (*N* = 30)				0.255
II	18	14	4	
III	12	7	5	
N0/N1				0.093
N0	33	22	11	
N1	11	4	7	
M0/M1				0.031
M0	33	23	10	
M1	11	3	8	
Rhabdoid cell				0.002
Not present	20	17	3	
Present	24	9	15	
FNCLCC grade				0.045
2	32	22	10	
3	12	4	8	
Vascular invasion				0.007
Not present	32	23	9	
Present	12	3	9	

*FNCLCC* French Federation of Cancer Centers

### Local Recurrence

**Fig. 1:**
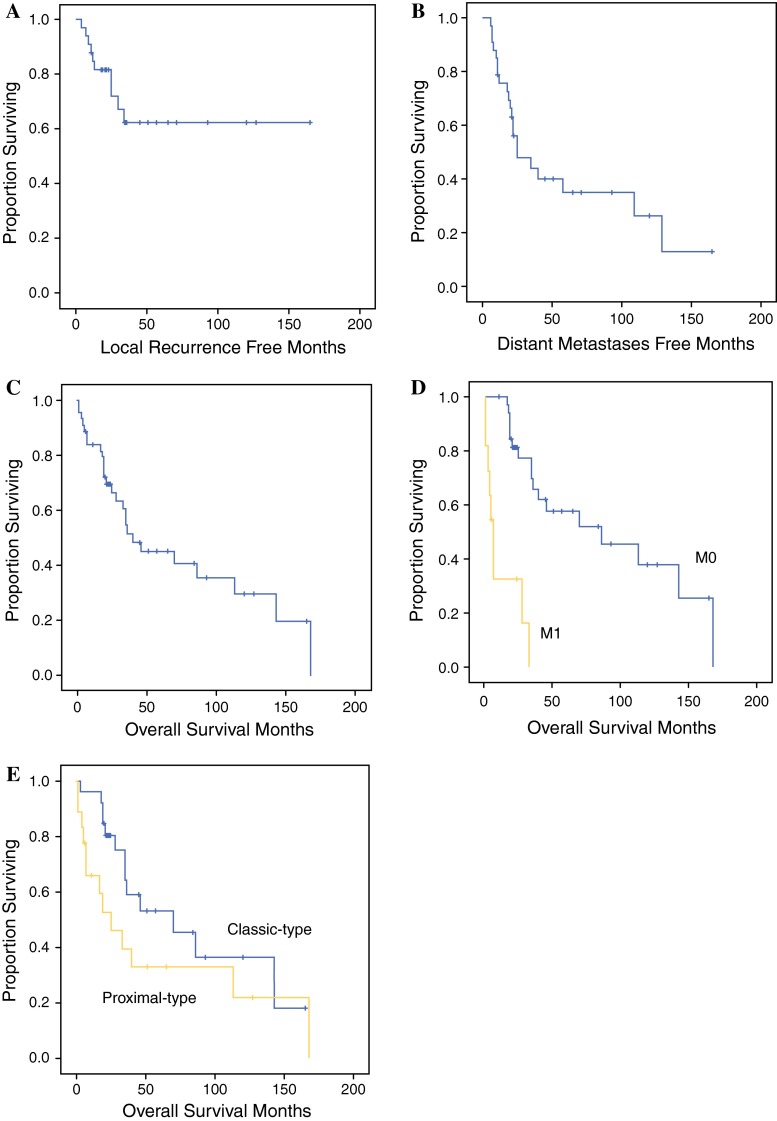
**a** Local recurrence-free survival rates for the 33 patients with M0 tumors were 82 and 62 % at 2 and 5 years, respectively. **b** Distant metastases-free survival rates at 2 and 5 years were 56 and 35 %, respectively. **c** Overall survival rates for the 44 patients overall were 70 and 45 % at 2 and 5 years, respectively. **d** Overall survival rates for the 33 patients with M0 tumors were 81 and 57 % at 2 and 5 years, respectively, and those for the 11 patients with M1 tumors were 33 and 0 %, respectively (*p* < 0.001). **e** Overall survival rates for the 26 patients with classic-type epithelioid sarcoma were 81 and 53 % at 2 and 5 years, respectively, and those for the 18 patients with proximal-type epithelioid sarcoma were 53 and 33 %, respectively (*p* = 0.124)

Among the 33 patients with M0 tumors who underwent curative surgery, local recurrences were noted in 10 (30 %). The LRFS rates at 2 and 5 years were 82 and 62 %, respectively (Fig. 1a). The median interval from surgery until the first local recurrence was 25 (range 4–165) months. Three of these ten patients developed both local recurrence and distant metastasis simultaneously.

Univariate analysis showed that a proximal tumor location (*p* = 0.020), superficial localization (*p* = 0.026), lymph node metastasis at presentation (N1) (*p* = 0.005), and a positive surgical margin (*p* = 0.036) were all correlated with LRFS. There was a tendency (*p* = 0.052) for an increased incidence of local recurrence in proximal-type ES relative to classic-type ES. Multivariate analysis showed that tumor depth (*p* = 0.040) and lymph node metastasis (*p* = 0.013) remained independent prognostic factors for LRFS (Table 2).

**Table 2:** Univariate and multivariate analysis of factors influencing local recurrence-free survival in 33 patients with M0 tumors

Variable	No. of cases	Univariate analysis			Multivariate analysis		
		2-year LRFS rate	5-year LRFS rate	*p*-Value	HR	95 % CI	*p* Value
Sex				0.348			
Male	24	83.1	69.3				
Female	9	77.8	46.7				
Age at presentation (years)				0.838			
≤30	11	72.7	72.7				
>30	22	86.4	57.6				
Location				0.020			NS
Distal	18	100	75.0				
Proximal	15	58.7	46.9				
Depth				0.026	0.260	0.072–0.941	0.040
Superficial	11	54.5	43.6				
Deep	22	95.2	71.4				
Tumor size (cm) (*N* = 30)				0.425			
≤5	20	75	60.0				
>5	10	88.9	71.1				
Stage (*N* = 30)				0.168			
II	18	88.9	72.7				
III	12	64.8	48.6				
N0/N1				0.005	5.714	1.436–22.739	0.013
N0	27	92.6	69.4				
N1	6	33.3	NA				
Morphological subtype				0.052			
Classic type	23	91.3	70.2				
Proximal type	10	58.3	43.8				
FNCLCC grade				0.265			
2	27	85	66.8				
3	6	66.7	44.4				
Surgical margin (*N* = 32)				0.036			NS
Negative	24	87.5	74.0				
Positive	8	62.5	31.3				
Adjuvant radiotherapy				0.547			
Done	6	83.3	83.3				
Not done	27	81.3	59.6				
Adjuvant chemotherapy				0.201			
Done	3	100	100				
Not done	30	79.7	57.0				

*NS* not significant, *HR* hazard ratios, *95 % CI* 95 % confidence interval

### Distant Metastasis

Distant metastases occurred in 32 (73 %) patients. The sites of metastasis at initial presentation included distant lymph nodes in 13 patients, the lung in 12 patients, lymph nodes and lungs in 4 patients, lung and muscle in 1 patient, the lung, liver and scalp in 1 patient, and bone in 1 patient.

Twenty-one (64 %) of the 33 patients with M0 tumors developed metastasis after a median period of 20 (range 6–122) months. The DMFS rates for these patients after 2 and 5 years were 56 and 35 %, respectively (Fig. 1b). Univariate analysis showed that only lymph node metastasis at presentation (*p* = 0.003) was a significant prognostic factor for DMFS, and this remained significant after multivariate analysis (*p* = 0.005) (Table 3).

**Table 3:** Univariate and multivariate analysis of factors influencing distant metastases-free survival in 33 patients with M0 tumors

Variable	No. of cases	Univariate analysis			Multivariate analysis		
		2-year DMFS rate	5-year DMFS rate	*p* Value	HR	95 % CI	*p* Value
Sex				0.321			
Male	24	42.6	37.3				
Female	9	77.8	31.1				
Age at presentation, years				0.847			
≤30	11	45.5	34.1				
>30	22	61.8	34.6				
Location				0.972			
Distal	18	54.5	29.2				
Proximal	15	58.2	41.6				
Depth				0.906			
Superficial	11	45.5	36.4				
Deep	22	61.2	32.6				
Tumor size (cm) (*N* = 30)				0.708			
≤5	20	50.0	31.2				
>5	10	56.3	42.2				
Stage (*N* = 30)				0.255			
II	18	55.6	41.7				
III	12	46.9	23.4				
N0/N1				0.003	4.101	1.508–11.155	0.006
N0	27	64.9	43.7				
N1	6	16.7	0				
Morphological subtype				0.937			
Classic type	23	55.2	29.4				
Proximal type	10	57.1	45.7				
FNCLCC grade				0.296			
2	27	61.3	39.4				
3	6	33.3	16.7				
Surgical margin (*N* = 32)				0.231			
Negative	24	56.7	45.4				
Positive	8	50.0	12.5				
Adjuvant radiotherapy				0.769			
Done	6	57.8	32.1				
Not done	27	50.0	50.0				
Adjuvant chemotherapy				0.239			
Done	3	54.8	31.3				
Not done	30	66.7	66.7				
Local recurrence				0.149			
Not occurred	23	53.7	53.7				
Occurred	10	60.0	10.0				

*NS* not significant, *HR* hazard ratios, *95 % CI* 95 % confidence interval

### Overall Survival

At the final follow-up, 14 (32 %) patients were alive without disease, 4 (9 %) were alive with disease, and 26 (59 %) had died of disease. OS at 2 and 5 years was 70 and 45 %, respectively (Fig. 1c). Univariate analysis showed that AJCC stage (*p* < 0.001), lymph node metastasis at presentation (*p* = 0.008), distant metastasis at presentation (*p* < 0.001), and histological grade (*p* = 0.019) were significant prognostic factors for OS. Multivariate analysis identified only distant metastasis at presentation as a significant prognostic factor (*p* < 0.001) (Table 4; Fig. 1d).

**Table 4:** Univariate and multivariate analysis of factors influencing overall survival of all 44 patients with epithelioid sarcoma

Variable	No. of cases	Univariate analysis			Multivariate analysis		
		2-year OS rate	5-year OS rate	*p* Value	HR	95 % CI	*p* Value
Sex				0.410			
Male	30	68.1	50.0				
Female	14	71.4	35.7				
Age at presentation (years)				0.704			
≤30	16	73.7	36.8				
>30	28	67.0	51.0				
Location				0.289			
Distal	20	80.0	52.5				
Proximal	24	60.7	38.7				
Depth (*N* = 43)				0.893			
Superficial	12	66.7	33.3				
Deep	31	73.1	53.6				
Tumor size (cm) (*N* = 39)				0.335			
≤5	22	72.7	46.2				
>5	17	63.1	36.1				
Stage (*N* = 41)				<0.001			NS
II	18	83.3	57.0				
III	12	72.7	48.5				
IV	11	32.7	0				
N0/1				0.008			NS
N0	33	78.0	57.2				
N1	11	45.5	11.4				
M0/1				<0.001	8.570	3.094–23.736	<0.001
M0	33	81.1	57.7				
M1	11	32.7	0				
Morphological subtype				0.124			
Classic type	26	80.6	53.2				
Proximal type	18	52.6	32.9				
FNCLCC grade				0.019			NS
2	32	74.3	56.5				
3	12	56.2	18.7				
Surgical margin (*N* = 32)				0.396			
Negative	24	78.3	56.9				
Positive	8	87.5	58.3				
Adjuvant radiotherapy (*N* = 33)				0.704			
Done	6	83.3	55.6				
Not done	27	80.8	58.0				
Adjuvant chemotherapy (*N* = 33)				0.414			
Done	3	66.7	66.7				
Not done	30	79.2	57.2				
Local recurrence (*N* = 33)				0.147			
Not occurred	23	81.6	62.7				
Occurred	10	80.0	50.0				

*NS* not significant, *HR* hazard ratios, *95 % CI* 95 % confidence interval

At 2 and 5 years, the OS of the 18 patients with proximal-type ES was 53 and 33 %, respectively. This was worse than the OS of the 26 patients with classic-type ES, i.e. 81 and 53 %, respectively (Fig. 1e).

## Discussion

ES is an aggressive but rare soft tissue tumor with severe consequences, even with currently available multimodal therapy. Risk factors for OS of ES patients have been reported to be large tumor size,^2^,^15^–^17^ deep-seated occurrence,^2^,^15^,^18^ proximal location,^2^,^8^,^9^ local recurrence,^15^,^18^ lymph node involvement,^2^,^10^,^18^ mitosis, necrosis, hemorrhage and vascular invasion.^2^,^16^,^19^ However, because of the rarity of ES, few data on its clinical behavior or the survival of affected patients are available. Recently, the proximal subtype of ES has been reported to have a poorer prognosis than the classical type.^6^,^7^,^9^–^11^ Rekhi et al. reported that proximal-type ES included a slightly higher proportion of M1 tumors (43 %) than classic-type ES (35 %), although the difference was not statistically significant (*p* = 0.61).^10^ In our present study of ES, the proximal type included a significantly higher proportion of M1 tumors (44 %) than the classic type (12 %) (*p* = 0.015) and tended to have a higher proportion of N1 tumors (39 %) than the classic type (15 %), although the difference was not statistically significant (*p* = 0.093).

It has been reported previously that 65–80 % of ES cases were classified as FNCLCC grade 2 tumors and 20–35 % as grade 3 tumors.^9^,^10^ In addition, it has been reported that proximal-type ES included a higher proportion of grade 3 cases (64–73 %).^9^,^10^ In addition, in our present study the proximal subtype included a significantly higher proportion of higher-grade tumors (67 %; *p* = 0.045). Histologically, proximal-type ES frequently shows rhabdoid features.^6^,^7^ Hasegawa et al. revealed that 14 (70 %) of 20 cases of proximal-type ES contained rhabdoid cells.^7^ We also observed a higher percentage of rhabdoid cells in proximal-type ES (83 %) than in classic-type (35 %) ES (*p* = 0.002). Proximal-type ES also showed a significantly higher rate of vascular invasion (50 %) than classic-type ES (13 %) (*p* = 0.007). To our knowledge, no previous report has mentioned the incidence of vascular invasion associated with ES subtypes. These results indicate that proximal-type ES has a more histologically aggressive nature than classic-type ES.

Deep-seated tumor localization has been reported to be an independent risk factor influencing LRFS.^12^ In our present study, a superficial location and lymph node metastasis were independent risk factors for local recurrence. Although this contrasts with previous reports, our finding could be explained by the higher number of superficial tumors in surgically more challenging sites, such as the perineal and inguinal regions, compared with other case series.^6^,^7^ In-transit metastasis is defined as a type of metastasis in which cancer spreads through a lymph vessel and begins to grow more than 2 cm away from the primary tumor before it reaches the nearest lymph node.^20^ Although in-transit metastasis has not been reported in ES, it may play a role in inducing local recurrence in soft tissue sarcomas, especially when there is a high potential for lymph node metastasis. This mechanism could explain the higher rate of local recurrence of N1 tumors in our present series.

ES has been reported to have a high rate of distant metastasis ranging from 40 to 57 %.^12^,^15^–^18^,^21^ The rate of 73 % recorded in the present study is higher than in previous reports; however, this could have been because a substantial number of cases in our series were either at a high tumor stage (N1 or M1 tumors, 39 %) or were recurrent cases (43 %) at initial presentation.^12^,^18^,^22^ The most common initial sites of distant metastasis in our series were the lung (45 %) and distant lymph nodes (43 %). Independent risk factors for DMFS have been reported to be large tumor size,^22^ deep-seated location,^18^ local recurrence, and regional lymph node metastasis.^12^,^18^ In our present series, the presence of lymph node metastasis was the only independent risk factor for DMFS.

The OS rates at 5 years for ES have been reported to be 32–78 %.^12^,^15^–^18^,^21^ In our series, the OS rate at 5 years (45 %) was relatively low but within the previously described range. We found that distant metastasis was the only independent factor predictive of a poor outcome. This would likely have been due to the limited number of ES patients, as well as the relatively short follow-up period (median 26.5 months).

It is still debatable whether prophylactic lymph node dissection is beneficial for ES patients.^12^,^15^,^17^,^21^^–^ 23 In the present study, lymph node dissection was indicated only when lymph node involvement was observed by diagnostic imaging. As lymphatic relapse was the most critical factor associated with poor outcome in both types of ES, it will be necessary to improve the detection and treatment of such lymphatic spread. Currently, we perform PET-CT examinations on all ES patients, and if lymph node involvement is suspected, we perform serial lymph node dissection and adjuvant radiation therapy if the resected node is histologically positive.

## Conclusions

We found that proximal-type ES had significantly higher clinicopathological aggressiveness than classic-type ES, and was associated with a proximal tumor location, a higher tumor stage, presence of rhabdoid cells, a higher tumor grade, and vascular invasion. Lymph node and distant metastases had the most critical impact on prognosis. Taking the rarity of this tumor into consideration, the number of patients in our series might have been too small to allow any definitive conclusion to be drawn. Therefore, further validation using an independent cohort of patients with ES is needed. Despite its slow growth, ES can be extremely aggressive when disseminated, and currently no useful treatments are available. Novel strategies are therefore needed to improve the survival of patients with these highly aggressive sarcomas.
